# Efficacy and safety of arthroscopic treatment for native acute septic arthritis of the hip joint in adult patients

**DOI:** 10.1186/s12891-021-04195-8

**Published:** 2021-04-01

**Authors:** Kensuke Fukushima, Yui Uekusa, Tomohisa Koyama, Yoshihisa Ohashi, Katsufumi Uchiyama, Naonobu Takahira, Masashi Takaso

**Affiliations:** 1 Department of Orthopaedic Surgery, Kitasato University School of Medicine, 1-15-1 Kitasato, Minami-ku, Sagamihara, Kanagawa 252-0374 Japan; 2 Department of Rehabilitation, Kitasato University School of Allied Health Sciences, 1-15-1 Kitasato, Minami-ku, Sagamihara, Kanagawa 252-0374 Japan

**Keywords:** Septic arthritis, Hip arthroscopy, Hip joint, Adult patient, Comorbidity

## Abstract

**Background:**

As septic arthritis is time-dependent and has a propensity for irreversible joint damage, early diagnosis and treatment are needed. Frequently, adult patients with septic arthritis cannot undergo invasive surgery because of comorbidities and a weakened immune system. Hip arthroscopic irrigation and debridement for native acute septic arthritis of the hip joint have been performed as the first choice of treatment for patients of all ages. This study aimed to assess the efficacy and safety of arthroscopic management for native acute septic arthritis of the hip joint in adult patients.

**Methods:**

Five adult patients (mean age, 46.2 years; all male) were retrospectively reviewed. Immediately after diagnosis, all patients underwent hip arthroscopic irrigation, debridement with synovectomy, and drainage. Partial weight-bearing was permitted once the C-reactive protein level normalised to < 1.0 mg/dl. Preoperative comorbidities, bacterial culture results, surgical complications, duration of hospital stay, time-to-confirmed normalisation of the C-reactive protein level, and recurrence incidence were evaluated.

**Results:**

All patients had comorbidities, and the cultured microorganisms differed among cases. There were no complications related to arthroscopic surgery. All patients achieved confirmed C-reactive protein normalisation within an average of 69.8 days, and there was no recurrence during the follow-up period (mean, 40.2 months; range, 16–60 months).

**Conclusion:**

Arthroscopic management for native acute septic arthritis of the hip joint is a safe and effective procedure in adult patients.

## Background

Septic arthritis is time-dependent, with a propensity for irreversible joint damage; hence, early diagnosis and treatment are needed [1, 2]. Native septic arthritis of the hip joint, which occurs more frequently in the paediatric population than in adults, has an incidence among adults ranging from 2 to 10 per 100,000 person-years [1]. Miyahara et al. have reported clinical and epidemiological characteristics of septic arthritis of the native hip for 7 years in the single institute of Orthopaedics and Traumatology [3]. They described that the frequency of septic arthritis of the hip joint reached a mean of just over two cases per year, and almost half of them were under 15 years old. Repeated aspirations, open arthrotomy, and arthroscopic treatment have been reported as surgical treatments for hip septic arthritis [4–6]. Historically, open arthrotomy remains the gold standard for treating septic arthritis of the hip joint [7]. Nonetheless, over the past few years, hip arthroscopy has become a common technique in both the diagnosis and treatment of hip disorders. Hip arthroscopic treatment has been described to provide improved visualisation, low rates of complications, and relatively rapid rehabilitation [8, 9].

Since 2011, we have performed hip arthroscopic irrigation and debridement for acute native hip septic arthritis as the first choice of treatment for patients of all ages. As adult patients with native hip septic arthritis tend to have comorbidities and a weakened immune system [3], we thought that surgical invasion should be minimized. Although arthroscopic treatment for septic arthritis might have many advantages for adult patients, few studies have specifically examined its efficacy and safety. Therefore, this study aimed to assess the efficacy and safety of arthroscopic management for native hip septic arthritis in adult patients.

## Patients and methods

### Ethics

We obtained approval from our Institutional Review Board (IRB) for this study, which was performed in accordance with the ethical standards laid down in the 1964 Declaration of Helsinki and its later amendments. The requirement for informed consent was waived because of the retrospective study design.

### Participants

We retrospectively enrolled 6 consecutive adult patients (aged > 20 years) diagnosed with acute native hip septic arthritis and underwent hip arthroscopic surgery at our centre between January 2011 to December 2018. Unfortunately, treatments for septic arthritis of hip joint (i.e.*,* treatment methods, surgical approach, and postoperative protocol including combined antimicrobial therapy) were not standardised in our institute before 2011. Therefore, we could not set the control group in this study. There were no patients diagnosed with native hip septic arthritis, and none underwent other treatments during the research. One of the 6 patients had bilateral hip septic arthritis, with joint destruction on one side; we performed resection arthroplasty and antibiotic spacer placement on the side with joint destruction and hip arthroscopic surgery on the other side. To assess the effectiveness of arthroscopic treatment alone, we excluded this patient from the study. Finally, the remaining 5 patients (5 hips) were included in the study.

All patients had a minimum of 1 year of follow-up, with an average of 40.2 (range, 16–60) months. Data regarding age, sex, preoperative comorbidities, the preoperative C-reactive protein (CRP) level, and the time period of symptoms prior to surgery were retrospectively collected from clinical records. All patients were male, and the average age was 46.2 (range, 37–62) years.

### Diagnosis of septic arthritis of the hip

All patients had complaints of severe groin pain and walking disturbances. After a thorough clinical evaluation, all patients underwent radiographic, ultrasonographic, and/or magnetic resonance imaging (MRI) examination of the hip joint and laboratory tests, including those for complete and differential blood counts and CRP level. We performed the hip joint aspiration in all patients with confirmed joint effusion based on ultrasonographic and/or MRI examination. In accordance with the study by El-Sayed et al. [10], a diagnosis of septic arthritis of the hip was established when the aspirate showed one or more of the following findings: 1) pus or turbid aspirate from the hip joint; and 2) a total synovial white blood cell count exceeding 50,000 cells per cubic millimetre, of which more than 90% are polymorphonucleocytes. In this case series, all operations were performed within 48 h of the diagnosis.

### Surgical procedure

All operations were performed under general anaesthesia by a single surgeon. During hip arthroscopy, patients were placed in a supine position upon a fracture Table. A well-padded perineal post was used, in addition to a carefully padded boot, with the heel firmly seated and secured. Gentle traction was applied to the legs bilaterally, and a spinal needle was used to establish an anterolateral portal over a guidewire under fluoroscopy. Two portals (anterolateral portal and mid-anterior portal) were used in all cases. Using a 4.0-mm 70-degree hip arthroscope for visualisation, irrigation and debridement of inflammatory synovium were performed for the entire hip joint. We harvested the inflammatory synovium to evaluate its bacterial content and for pathological examination. Finally, a 3.5-mm continuous negative suction drain was left via the mid-anterior portal.

All patients were permitted to move using a wheelchair the day after surgery. Once CRP normalisation to less than 0.5 mg/dl was confirmed, suction drainage was permitted to remove less than 10 ml/day of fluid. We have experienced many patients who underwent hip arthroscopic surgery for a labral tear, and hip arthritis increased the CRP level after permitting weight-bearing. Therefore, we permitted partial weight-bearing at the time of CRP normalisation, and once the CRP level did not increase with partial weight-bearing, full weight-bearing was permitted.

The duration of antimicrobial therapy was based on the clinical response, causative microorganisms, and CRP level. Intravenous antibiotics were started immediately after sample harvesting and administered for a minimum of 2 weeks, followed by oral antibiotics for an additional 3 months.

### Evaluation of the treatment

Data regarding the causative microorganisms, investigated by macrobacterium examination during the surgery, were recorded. Surgical complications were investigated. Additionally, the duration of the hospital stay and the time-to-confirmed CRP normalisation were determined from medical records. Furthermore, we evaluated the incidence of recurrence during the follow-up period.

## Results

Preoperative patient data are shown in Table 1. All patients had comorbidities. In addition, 3 patients (60%) had other infections, including pulmonary tuberculosis. We performed open drainage to treat an iliopsoas abscess combined with hip septic arthritis in patient 1. The mean preoperative CRP level was 18.29 (range, 5.35–29.09) mg/dl. The mean time period of symptoms prior to surgery was 10.2 (range, 3–21) days.

**Table 1:** Preoperative patient data

Patient No.	Age (years)	Sex	Comorbidities	Preoperative CRP (mg/dl)	The time period of symptoms prior to surgery (days)
1	41	Male	SMA thrombosis, iliopsoas abscess	5.35	21
2	62	Male	Prostate cancer, multiple bone metastasis	14.01	11
3	37	Male	Pulmonary tuberculosis	20.49	4
4	40	Male	Pneumonia	22.51	12
5	51	Male	Type 1 Diabetes	29.09	3
Average	46.2			18.29	10.2

*CRP* C-reactive protein, *SMA* Superior mesenteric artery

Synovial cultures yielded positive findings in 4 patients (80%); however, the causative microorganisms were different in each case (Table 2). In case 1, the patient had been administered antibiotics by an internal medicine physician before coming to our clinic, and no microorganisms were identified in this case.

**Table 2:** Causative microorganisms

Patient No.	Culture results
1	None
2	*Staphylococcus species*
3	*Streptococcus agalactiae (Group B)*
4	*Haemophilus influenzae*
5	*Staphylococcus aureus (MSSA)*

Treatment evaluation data are shown in Table 3. No apparent complications were noted during the treatment. All patients completed postoperative treatment as per our postoperative protocol. Although some patients required treatment for comorbidities, all patients achieved remission of their complaints regarding hip pain and CRP normalisation. The average time-to-confirmed CRP normalisation was 69.8 (range, 11–217) days. The average duration of hospital stay was 34.4 (range, 20–56) days.

**Table 3:** Treatment evaluation

Patient No.	Surgical complications	Time-to-confirmed CRP normalisation (days)	Duration of hospital stay (days)	Recurrence
1	None	11	20	None
2	None	17	26	None
3	None	217	44	None
4	None	18	26	None
5	None	86	56	None
Average		69.8	34.4	

*CRP* C-reactive protein

During the follow-up period, there were no cases of infection recurrence or progression of osteoarthritis.

## Discussion

In the present study, we assessed the efficacy and safety of arthroscopic management for hip native septic arthritis in adult patients. All patients achieved confirmed CRP normalisation within an average of 69.8 days, and none experienced recurrence. Furthermore, despite the presence of severe comorbidities in all patients, we did not encounter any complications during treatment.

Hip arthroscopy currently provides excellent visualisation of not only the articular surfaces of the hip joint but also the peri-trochanteric, or extra-articular, space around the hip. Hip arthroscopy is considered a very effective method for examining and treating joint disorders, and its use is widely increasing [8]. Traditionally, open arthrotomy has been the most common surgical approach for septic arthritis of the hip joint [4, 6]. However, although aggressive debridement can be performed, hip function decreases after open arthrotomy. In addition, operative invasiveness is much greater in open arthrotomy than in arthroscopic surgery. It has been reported that the risk factors for septic arthritis, especially in adults, include an immunodeficient state, rheumatologic diseases, skin infections or trauma, and corticosteroid injections. In the present series, all patients had severe comorbidities and/or combined infections at other sites [2–4]. Khazi et al. assessed differences in short-term complications between patients treated with open arthrotomy or arthroscopy for native hip septic arthritis and identified the risk factors associated with a return to the operating room [11]. According to their data, patients who underwent arthroscopy had fewer total complications, and preoperative septicaemia, or septic shock, was a significant risk factor for a return to the operating room. De SA et al. performed a systematic review to assess the efficacy of hip arthroscopic management for septic arthritis in the hip joint. In their review, no major or minor complications were reported [7]. Similarly, our results suggest that arthroscopic management is a safe option for treating septic arthritis in adults, even in those with risk factors because of severe comorbidities.

Septic arthritis in the native hip joint occurs less frequently in the adult population than in the paediatric population; it is considered a rare disorder. Accordingly, there are only a few reports on the clinical results of arthroscopic treatment for native hip septic arthritis in adult patients. Yamamoto et al. described such clinical results in 4 adult cases [12]; although arthroscopic treatment was performed an average of 36 days after the initial onset of symptoms, infection eradication and joint preservation were achieved. Additionally, Lee et al. reported a case series with 9 adult patients [13]; among these, only one patient experienced septic arthritis relapse. In the present case series, all patients achieved infection eradication without recurrence during follow-up, even though the average follow-up period was longer in the present study than in the previous studies.

Arthroscopic treatment has advantages over open surgery in that it allows more detailed debridement of necrotic tissue and purulent material, with preservation of anatomical structures (such as muscles and ligaments) around the hip joint. Additionally, arthroscopic treatment allows sufficient irrigation via a circulating pump system and direct inspection of cartilage in both the acetabular and femoral sides, with minimal operative morbidity [13–15]. Considering the volume of soft tissue around the hip joint, these advantages might result in greater efficacy in adult patients than in paediatric patients. As arthroscopic surgery is less invasive than open surgery, the decision to proceed to early surgical intervention might be easier. However, it should be mentioned that hip arthroscopy requires several special instruments and has a steep learning curve [16–18].

There are several limitations to this study. First, as the main limitation, the study design had a few case report series, and there was no control group. Second, we conducted this study retrospectively. Third, all operations were performed by a single experienced surgeon, and the time period of symptoms was relatively short. Overall, other factors might influence the results, which could not be assessed in this study. Since native hip septic arthritis in the adult population is rare, further multi-centre studies might be needed.

## Conclusion

Arthroscopic management for native acute septic arthritis of the hip joint in adult patients is a safe and effective procedure.

## Data Availability

The datasets supporting the conclusions of this article are included within the article. The raw data can be requested from the corresponding author.

## Abbreviations

IRB: Institutional Review Board
CRP: C-reactive protein
MRI: Magnetic resonance imaging
